# Passive Monitoring of Crowd-Level Cough Counts in Waiting Areas produces Reliable Syndromic Indicator for Total COVID-19 Burden in a Hospital Emergency Clinic

**DOI:** 10.21203/rs.3.rs-3084318/v1

**Published:** 2023-06-26

**Authors:** Forsad Al Hossain, Tanjid Hasan Tonmoy, Sri Nuvvula, Brittany P. Chapman, Rajesh K. Gupta, Andrew A. Lover, Rhoel R. Dinglasan, Stephanie Carreiro, Tauhidur Rahman

**Affiliations:** 1 Halıcıoğlu Data Science Institute, University of California, San Diego, San Diego, CA, USA.; 2 Department of Emergency Medicine, UMass Chan Medical School, Worcester, MA, USA.; 3 College of Information and Computer Sciences, University of Massachusetts Amherst, Amherst, MA, USA.; 4 School of Public Health & Health Sciences, University of Massachusetts Amherst, Amherst, MA, USA.; 5 Infectious Diseases and Immunology, University of Florida, Gainesville, FL, USA.

**Keywords:** Ambient Sensing, Cough Counting, Syndromic Surveillance, Emergency Medicine, Respiratory Illness

## Abstract

Syndromic surveillance is an effective tool for enabling the timely detection of infectious disease outbreaks and facilitating the implementation of effective mitigation strategies by public health authorities. While various information sources are currently utilized to collect syndromic signal data for analysis, the aggregated measurement of cough, an important symptom for many illnesses, is not widely employed as a syndromic signal. With recent advancements in ubiquitous sensing technologies, it becomes feasible to continuously measure population-level cough incidence in a contactless, unobtrusive, and automated manner. In this work, we demonstrate the utility of monitoring aggregated cough count as a syndromic indicator to estimate COVID-19 cases. In our study, we deployed a sensor-based platform (*Syndromic Logger*) in the emergency room of a large hospital. The platform captured syndromic signals from audio, thermal imaging, and radar, while the ground truth data were collected from the hospital’s electronic health record. Our analysis revealed a significant correlation between the aggregated cough count and positive COVID-19 cases in the hospital (Pearson correlation of **0**.**40**, p-value < **0**.**001**). Notably, this correlation was higher than that observed with the number of individuals presenting with fever (***ρ* = 0**.**22**, ***p* = 0**.**04**), a widely used syndromic signal and screening tool for such diseases. Furthermore, we demonstrate how the data obtained from our *Syndromic Logger* platform could be leveraged to estimate various COVID-19-related statistics using multiple modeling approaches. Our findings highlight the efficacy of aggregated cough count as a valuable syndromic indicator associated with the occurrence of COVID-19 cases. Incorporating this signal into syndromic surveillance systems for such diseases can significantly enhance overall resilience against future public health challenges, such as emerging disease outbreaks or pandemics.

## Introduction

The act of coughing is part of the body’s defense system that uses automatic reflex mechanisms to clear airways of foreign substances, irritants and excessive mucus. Cough and related complaints have been recognized as a cardinal symptom or syndromic signal of respiratory illnesses, such as the SARS-CoV-2, influenza and influenza-like illness, asthma, chronic obstructive pulmonary disease (COPD), and lung cancer. Recent progress with cough assessment has led to improved diagnosis and more effective disease management for individual patients [10, 18]. However, cough-related signals have been largely absent in population-level disease monitoring and public health surveillance. This is primarily due to lack of efficacious, scalable and contactless sensing infrastructure to gather cough event information (e.g., total occurrences at a location) from specific target populations.

In this work, we present an ambient sensing platform to capture different syndromic signals (e.g., daily cough counts) from the emergency department (ED) waiting room of a large hospital within a large metropolitan area. The deployed sensor platform *Syndromic Logger* is the next-generation contactless sensing platform[3]. The *Syndromic Logger* platform captures non-speech audio with a microphone array for the detection of cough and speech events. With a built-in thermal camera, it can continuously capture thermal video to detect number of people in the hospital waiting areas (as a measure of waiting room crowd size). Lastly, with a ultra-wideband radar sensor, the platform can also capture human movements. In this work, we specifically focus on the significance of aggregated cough count as a syndromic signal for COVID-like illnesses. We collected sensor data over a period of four months and obtained ground truth data from electronic health record systems (EHR). Our results demonstrate that aggregated cough count is a strong indicator of total COVID burden within the hospital. Compared to aggregated fever count, a widely used syndromic indicator for population-level epidemiological models of respiratory diseases, we found stronger associations between total cough count and total COVID-19 case counts. Moreover, we show that the automatically captured syndromic data from *Syndromic Logger* in a waiting room can be used to develop a regression model for daily counts of COVID-19 cases in a hospital emergency clinic. Inclusion of such automatic syndromic signals/- data using historical data and manually extracted information from EHRs in a regression model boosts the performance of daily COVID-19 case prediction. Overall, the paper highlights the capability of automatic, passive and contactless syndromic sensing for population-level COVID-19 burden monitoring in a hospital setting.

## Primer on Syndromic Surveillance and the Need for Contactless Syndromic Sensing

As the world manages the staggering global public health crisis of COVID-19 and begins to move toward a “new normal”, our vulnerabilities to another out-break of SARS-CoV-2 (or an equally devastating pathogen) are ever apparent. The ability to keep society safe and functional in the event of a resurgence is of paramount importance [23]. The state-of-the-art disease monitoring and public health surveillance by the US Center for Disease Control and Prevention (CDC) primarily relies on aggregated reports from sentinel reporting sites including hospitals and selected outpatient clinics. However, there exists a substantial time lag in the reporting of such data (e.g., 7–14 days reporting lag time for influenza-like illness) [12, 17]. The lack of real-time information on the infection dynamics and symptom dynamics of the target population is a fundamental gap that limits our ability to monitor and forecast disease trends to mobilize early interventions. Smart and connected syndromic surveillance with state of the art sensor systems to capture objective syndromic signals (e.g., cough, fever) unobtrusively from target population to enhance predictive intelligence and pandemic resilience strategies represent a prime opportunity. Such a syndromic computing framework can help to mobilize a rapid public health response, limiting the spread of infection, and consequent morbidity and mortality.

While syndromic surveillance has been long utilized in public health, it is an emerging area in computational epidemiology, due to the accessibility of high-resolution data sources. This paradigm aims to gather general symptom-related information (prior to any clinical diagnosis) of an infectious disease to allow for rapid responses. Several recent works aimed to achieve such goals especially in the context of the recent COVID pandemic using both active and passive monitoring of syndromic signals. Examples of active monitoring include self- reported body temperature and presence of symptoms relevant to COVID using a mobile application [6]; symptom and demographic data from people searching about COVID symptoms through a chatbot [24]; symptom and testing data using internet and phone surveys [11]; and voluntarily-reported symptom data [16]. These approaches require active participation from the target population, may be clinically unreliable due to self-reporting, and may not capture subsets of vulnerable populations (e.g. older and less technology savvy demographics). Examples of passive monitoring include monitoring of emergency room activity in hospitals [2, 9], monitoring occupancy, and crowd size [20]. These approaches might be subject to reporting delays, require active human effort (e.g. manually measuring and logging data) and may fail to capture objective and relevant syndromic signals. To address these limitations, it is desirable to capture crowd level syndromic signals in a passive, contactless and automated manner. In addition, for genuine public health impact, syndromic surveillance platforms need to be scalable and to sprovide objective and real-time metrics informative of the total burden of specific disease burden in the target population or community.

## Methods

### Study Overview

This study was reviewed by the Institutional Review Board (IRB) at the University of Massachusetts Amherst. We deployed our device in the emergency department (ED) waiting room of a large, tertiary-care, academic medical center with a census of over 135,000 emergency visits per year. The ED operates 24 hours a day, seven days a week. The waiting room space is occupied by hospital staff (nurses, patient care technicians, and security personnel), adult and pediatric patients, and accompanying visitors during the triage process. On busier days, patients and visitors may spend several hours in the waiting room prior to a bed becoming available in the main treatment area, which is in a separate location from the waiting area. We placed our sensor in the ED waiting room from August 1, 2021 to November 30, 2021, and then again from March 1, 2022 to April 30, 2022 for continuous syndromic data collection. Within the emergency department waiting area, the specific location for our device placement was determined based on the availability of power outlet, maximal capture area of thermal camera with its limited field of view, and high signal to noise ratio audio data recording. Routine checks of the device were performed during the data collection process to ensure proper positioning in the waiting room.

### Syndromic Surveillance Platform

**Fig. 1:**
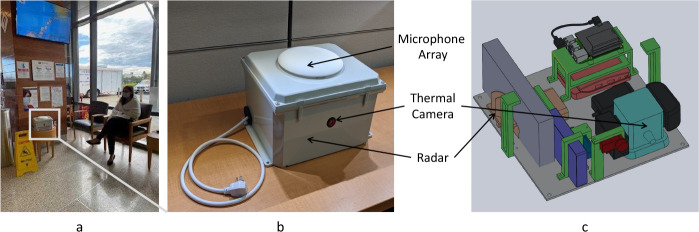
a) Deployment location sample with an actor simulating the patient’s sitting location; b) Top view of the ***Syndromic Logger*** device from outside c) 3D-render of internal components of the ***Syndromic Logger*** device

Our *Syndromic Logger* platform (depicted in Figure 1) integrates a wide range of sensors and hardware components. It also incorporates a software system that leverages multiple machine learning models and ensures secure data storage. In the following subsections, we provide detailed descriptions of the various components and functionalities of the platform.

### Hardware

Our ***Syndromic Logger*** platform includes the the following hardware modules:

- ReSpeaker Microphone Array V2.0: A microphone array containing four microphones (http://wiki.seeedstudio.com/ReSpeakerMicArrayv2.0/).
- SEEK CompactPRO: An inexpensive thermal camera (https://www.thermal.com/compact-series.html/) that can be used for people counting using thermal video.
- Intel Neural Computing Stick: A hardware platform (https://software.intel.com/en-us/neural-compute-stick) that is used for accelerating Deep Neural Network computation.
- PulseON 440 UWB radar: A radar sensor for monitoring movements and motions in the deployment area (https://fccid.io/NUF-P440-A/User-Manual/User-Manual-287844).
- Raspberry PI: A fully Linux-based embedded platform used for managing the sensors and processing and storing the data securely in real-time.

### Software system

#### Audio processing

We developed our speech and cough recognition classifier using the dataset and model architectures described in [3]. For the construction of the cough and sneeze recognition model, we utilized the dataset sourced from [1], which was also used by the authors of [3]. To ensure consistency, we adhered to the data augmentation procedure outlined in [3] and generated corresponding training, testing, and validation datasets.

For cough detection, we used the VGGish model[8]. The VGGish was trained on AudioSet data[5]. AudioSet is a massive collection of Youtube videos containing 10 seconds of weakly-labeled video segments; it includes 70 million videos collected from youtube. The VGGish architecture is a variant of VGGNet architecture [21], with only the last layer changed and the Local Response Normalization (LRN) layers changed with BatchNormalization layers [8]. To use the model with audio signals, we used the same dataset and augmentation pipeline from FluSense authors[3] and converted each 1 second long audio snippet into log-mel spectrograms of size 96 × 64.

Table 1 shows the performance of our cough model in different environments compared to the model used in prior work [3]. We considered three environments: testing data without any augmentations, testing data with speech inserted in it, and testing data with hospital noise collected from YouTube. We also used the same dataset as [3], which is the real-world hospital data containing cough and non-cough sounds. The list of non-cough sounds includes sounds that are abrupt in nature, similar to cough, such as door-slamming and dropped objects. The original model in [3] had many false positives for these sounds. As evident in table 1, our model works robustly in all testing conditions and outperforms the original model in [3], achieving the best results during testing with real-world data samples even in the case when the training data did not include any such real world data.

For deployment, we first converted the models to 16-bit floating point precision model and then deployed the optimized model using OpenVino (https://www.intel.com/content/www/us/en/developer/tools/openvino-toolkit/overview.html) framework. We used Intel NCS (version 2) to process the audio data stream in real time.

#### Thermal data processing

We captured thermal video data from SEEK Thermal Pro camera. The resolution of our camera was 320×240 pixels, and our capture frequency was 5 frames per second.

Similar to the authors of [3], we used a trained Faster-RCNN model [19] to detect the number of people from the video data. With the trained model, extract bounding boxes containing people and then use the bounding box areas to extract various features from the video data.

#### UWB Radar data processing

We used PulseON 440 UWB radar in the monostatic mode for collecting UWB radar data. The radar operates within bandwidths of 3.8 to 4.8 GHz with a center frequency of 4.3 GHz. It has a transmitter antenna that emits millions of very short duration pulses. These pulses are reflected both by stationary objects and moving objects. A co-located receiver antenna receives these backscattered signals, and multiple scans of such received signals are stacked together to form an image representation, which is also known as a “radargram”. One dimension of the image corresponds to different range bins, which are generated by collecting reflected signals with varying time of flight. This time of flight, multiplied by the speed of light, estimates the round trip distance to a specific bin. The second dimension represents time, often referred to as slow time. Objects or humans in motion exhibit distinct signatures that fluctuate or traverse different range bins over the course of time.

To extract movement-related features, we removed the static clutter part of the radargram caused by static objects from the radargram. To achieve this, we first calculate the mean for each range bin of the radargram and then subtract the mean from each radargram. Afterwards, the radargram contains only the signals caused by movements from non-static elements in the environment (mostly people). After clutter removal, we apply the Fast Fourier Transform to each range bin to get a Fourier spectrum from each range bin.

#### System security and preservation of privacy

Finally, to store all of the saved data in a secure manner, we used a 2-phase encryption scheme to store the data in a hard drive attached to our system. In the first phase, a random key is generated, and this key is used for encrypting captured data (i.e., audio snippet, video clip, or radar data) using symmetric encryption schemes. Using symmetric encryption ensures that our CPU load is low during this data chunk encryption process. After that, this randomly generated key itself gets encrypted with a public key, and it gets stored with the encrypted captured data. To decrypt data snippets, at first, we use a private key that is only available to us to decrypt the symmetric encryption key that was stored with the data snippet, and then we use that symmetric key to decrypt the stored sensor data. This 2-phase encryption scheme ensures that our data is secure, and in the case of theft or vandalism, data in our platform remains inaccessible without the private key.

As the US Federal Health Insurance Portability and Accountability Act (HIPAA) of 1996 forbids collecting any privacy-sensitive data within the scope of a non-HIPAA compliant framework, we explicitly did not save any speech data during our deployment. To omit these speech data from our saved dataset, we built the same model as the authors of [3], which was shown to be very effective in a real-world deployment. During runtime, we detected whether we had speech content in each 1-sec slice of audio data. We only saved data for future analysis if it didn’t contain any speech. Otherwise, we skipped saving the data to preserve user privacy.

### Ground Truth Data Collection

For ground truth data, aggregate daily data points were abstracted from electronic health record (EHR) and managed using REDCap (Research Electronic Data Capture) tools hosted at the University of Massachusetts Chan Medical School ([7]). The ground truth data includes daily information about the ED patient volume, wait time, number of viral tests (COVID and non-COVID) ordered, and the number of positive viral tests (COVID and non-COVID).

Of note, COVID testing is done for a variety of reasons in the hospital (e.g. for diagnosis in symptomatic patients, to screen asymptomatic patients before a procedure, or before entry into a group care setting). For the purpose of this study, we excluded COVID tests ordered on asymptomatic patients for screening purposes, as their volume was more likely related to hospital policy and other factors as opposed to changes in community prevalence of SARS-CoV-2 infections. Symptomatic testing includes both immunoassays (commonly referred to as “Rapid” tests) and polymerase chain reaction-based (PCR) assays. We collected the following ground truth data fields.

- **Number of People**: This number of patient visits the emergency department (ED) for a given day.
- **Average Wait time**: The average time an ED patient waited prior to being placed in a bed in the main treatment area
- **Any COVID Test**: The total number of COVID −19 tests ordered on symptomatic ED patients. This number includes COVID tests as ordered within a Respiratory Viral Panel (RVP) and standalone COVID tests.
- **Positive COVID**: The total number of positive symptomatic ED COVID-19 tests. This includes tests ordered as part of Respiratory Viral Panel (RVP) and non-RVP).
- **Positive Flu**: The total number of positive ED Flu Type-B using RVP testing methods.
- **Positive RSV Results**: The number positive ED RSV (Respiratory Syncytial Virus) tests using RVP tests.
- **Fever (**> 38*C*°**)**: The total number of unique ED patients that had a fever recorded on their triage vital signs: fever is defined as temperatures greater than or equal to 38° degrees Celsius.

#### Feature Extraction

In our study, we utilized our *Syndromic Logger* platform to gather syndromic signal data from audio, thermal, and UWB radar. We extracted the following features from the collected data.

#### Audio-based Features

We process ambient audio using an on-device machine learning model and used the following features for analysis:

- **Total cough Count** (Total cough): The total daily cough count detected by our sensor platform.
- **Total Speech Count** (Total Speech): This feature is the total number of speech snippets detected by our system on a daily basis.

#### UWB Radar-based Features

The Ultra-Wideband (UWB) radar data captures movement-related features from the radar signals as described earlier. We extracted the following features for each day.

- **Filter Bank Features**: Human movement generates relatively low frequency responses and broadband radar signals, while machinery such as a fan and air conditioning units generate high frequency monotonic signals. Consequently, the spectral analysis of the radar signal can allow us to look at different types of movements captured by the UWB radar. We take a filter bank approach to quantify the energy in different frequency ranges or filter banks. To be specific, the cut-off frequencies associated with the filter banks are respectively 0.25–0.50 Hz, 0.5–0.75 Hz, 0.75–1.0 Hz, 1.0–1.25 Hz, 1.25–1.5 Hz, 1.50–1.75 Hz, 1.75–2.0 Hz, 2.0–2.25 Hz, 2.25–2.5 Hz, 2.5–2.75 Hz, 2.75–3 Hz. We observed in our analysis that human movement is associated with low frequency filter banks.
- **Total Energy**(*tot energy*): The total energy in the radar signals capture the total human movement related activities that occur in the waiting room. A crowded waiting room where the occupants are moving will consequently increase the magnitude of this total energy feature.
- **Standard deviation of energy per bins** (*std energy*): We also extract the standard deviation of total average energy in different range bins which captures a measure of waiting room crowd dispersion.

#### Thermal Camera-based Features

We captured thermal video data from SEEK Thermal Pro camera. The resolution of our camera was 320×240 pixels, and our capture frequency was 5 frames per second. Similar to our prior work outlined here [3], we used a trained Faster-RCNN model [19] to detect the number of people from the video data. With the trained model, extract bounding boxes containing people and then use the bounding box areas to extract various features from the video data.

- **Person Time** (Person-Time): Upon detecting the number of people in the waiting room based on the total number of bounding boxes in every frame (captured at a rate of 5 frames per second), we aggregate the total number of bounding boxes for the entire day to derive the ”Person Time” feature. This feature is a fairly standard feature used in syndromic models which can inform the model with crowd-level metric.
- We did not extract body temperature feature from the thermal camera. This is primarily because of the relatively high temperature measurement error. While this thermal camera proves to be sufficient to reliably estimate human body bounding boxes, high-fidelity human body temperature estimation requires a sophisticated (expensive) thermal camera which was not aligned with the needs for a routine health surveillance platform.

## Results

### Correlation Analysis: Syndromic Signals and Population-level Disease Metric

Table 2 presents the Pearson correlation coefficients between different sensor-based syndromic signals captured by our device and ground truth population-level disease burden metric extracted from the hospital’s EHR system. which includes the number of people counted per day and total positive COVID, Flu, and RSV patient count.

### Key Observation 1: Total daily cough count has statistically significant correlation with positive COVID-19 case count.

**Fig. 2:**
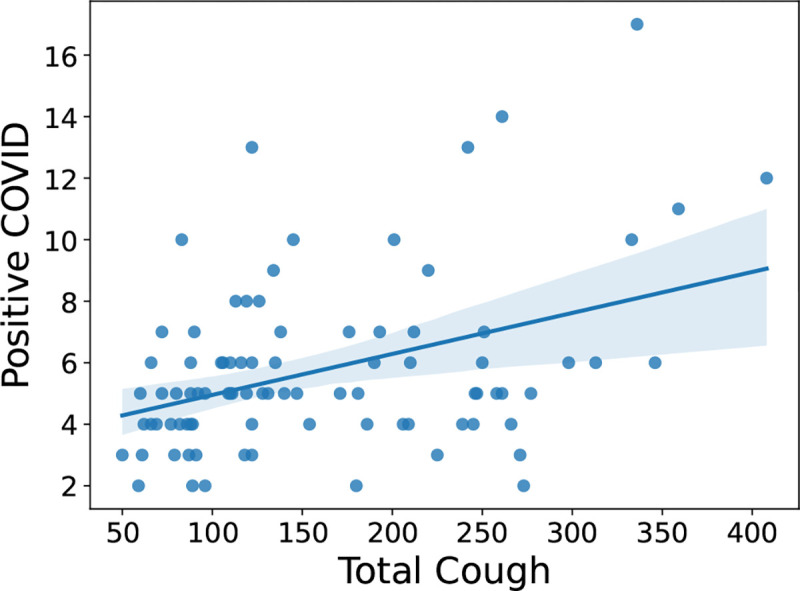
Scatter plot showing correlation between Total cough and positive COVID

When examining the correlation coefficients, we observe the most significant correlation between total daily COVID positive patients and the total number of coughs captured by our device (.40 with a p-value of 0.0001). This relationship is visually depicted in Figure 2. Moreover, we also observe relatively high correlations between total cough and total daily positive RSV patients (0.27 with a p-value of 0.01). However, we did not observe any strong correlation with positive influenza count. This can be attributed to the fact that flu has generally more variable with the presence or absence of respiratory symptoms (e.g. some people have only gastrointestinal symptoms or muscle aches without cough).

To quantify time series trends with adjustment for serial correlation, multivariable models with negative binomial distribution and robust (Huber-White “sandwich” errors) were used. Additional sensitivity analyses were performed using bootstrapped errors with 2,000 replicates. All exploratory factors with *p* < 0.20 were taken into multivariable models, with model building guided by lowest AIC/BIC. “Day of week” was forced into all models regardless of significance. All tests were t-tailed, with *α* = 0.05. Analyses used Stata 17 (College Station, TX). To quantify the association between sensor-based metrics and positive SARS-CoV-2 tests, multiple cough-based measures were assessed, including “total coughs;” “total coughs” normalized to ln(low-band radar signal, 0.25–0.50 Hz); and “total coughs” normalized to thermal-sensor person-time.

The results from multivariate time series models are shown in Table 4. For each 13% increase (95% CI: 6% to 20%) in captured cough sounds (standardized to the lowest frequency radar bound output) there was a one-unit increase in positive SARS-CoV-2 tests, with adjustments for day-of-week.

### Key Observation 2: Total number of people visiting ED waiting room and total daily RSV count has a statistically significant correlation with positive SARS-CoV-2 tests.

Table 3 shows the correlation between the ground-truth variables. We can see high correlation coefficients between daily positive COVID patient count and the number of patients with positive RSV (0.38 with a p-value of 0.0003). This could be because the viruses peaked in the community around the same time and because people were co-infected [14, 22]. Additionally, we can see that the number of people, and the number of positive COVID patients has a correlation coefficient of 0.22. Another important metric, fever > 38°*C*, which is often used an screening measure for detecting COVID, showed a stronger correlation with the number of people in the waiting room compared to the number of positive COVID cases. Specifically, we found correlation coefficient of 0.22 between fever and total COVID case count. In contrast, the correlation with number of people is 0.45, which is much higher than the value for positive COVID tests.

### Key Observation 3: The syndromic features extracted from radar (e.g., 0.0–0.25 Hz filter band energy feature) achieves statistically significant correlation with daily average wait time extracted from EHR. However, the Person-Time feature extracted from the thermal camera failed to show any statistically significant relationship as illustrated in figure 3.

This can be explained by the fact that the radar has a significantly greater sensing coverage area and can capture any movement in circle with 5 meter radius. The thermal camera, on the other hand, can capture thermal images with a 320×240 pixel resolution and a 32° field of view. The limited field of view impact the thermal camera’s ability to capture the entire waiting room crowd and consequently the estimated Person-Time from the limited field of view thermal images fail to capture the waiting room crowd behavior such as average wait time.

### Modeling COVID burden using sensor-based features

We aim to demonstrate the effectiveness of incorporating cough count as a feature in modeling COVID burden in the hospital, emphasizing its value as a syndromic signal. First, we develop baseline models that do not incorporate any sensor-based features. Subsequently, we show the utility of sensor-based features, particularly cough count, in enhancing the modeling of COVID-related statistics compared to the baseline. We evaluate the models using Mean Average Error (MAE) and correlation coefficient. Table 5 summarizes the different feature sets we use for the models. We chose the weekday index, a categorical variable that represents the day of the week, as our first baseline feature set B1. The remaining baseline feature sets (B2-B4) include positive COVID case counts from up to three previous days, enabling models to perform autoregression with historical data. We explored different models with baseline feature set and random forest yields the best performance when trained on baseline feature sets. We can see the results in table 6. Among all the baseline feature-based model, the random forest trained on B4 feature set achieves the best performance with a correlation coefficient *ρ* of 0.13 and an *MAE* of 2.14. However, compared to the sensor-informed syndromic feature-based models, the baseline models achieves a significantly lower performance.

To assess the performance of sensor-based features compared to the baseline features, we selected three highly correlated input features for modeling the count of positive COVID cases: ‘Total Cough’, ‘Total Speech’, and ‘std energy’ (correlation coefficients of 0.40,0.20, and 0.15, respectively). We conducted experiments employing various models, including Linear models (Linear Regression, Poisson Regression), Tree based models (Random-Forest, Gradient boosted trees), and Support Vector Machines with an ‘RBF’ kernel. The Gradient-Boosted trees achieve the best results in terms of Pearson Correlation *ρ* (0.45), while the Support Vector Regression (SVR) model achieves the best *MAE* of 1.66. Overall, models using syndromic features (i.e., S1 and S2) from the Syndromic Logger platform substantially outperform the models using baseline features for the task of predicting the total COVID positive patient count.

In addition to the total number of COVID patients per day, we also modeled the prevalence of COVID as a percentage. To normalize the COVID count, we considered two scenarios: (a) the percentage of COVID-positive cases among the number of ED waiting room visitors, and (b) the percentage of COVID-positive cases among the total number of tests conducted. In both cases, our models utilizing sensor-based syndromic features exhibited significant improvements over the baseline models. For predicting the ratio of Total Positive COVID cases to the total number of people, our best model achieved a correlation coefficient of 0.53. It is significantly better compared to the baseline, which reached a correlation coefficient of 0.25 (with B2 feature set). Similarly, for predicting the percentage of COVID-positive patients among the total number of tests performed, our best model achieved a correlation coefficient of 0.43, in contrast to the baseline results of a correlation coefficient of 0.02 (with B4).

### Comparing with Body temperature based surveillance

Traditionally, ‘Fever (> 38*C*°)’ is used as a biomarker for screening respiratory illnesses. However, in our data, the number of patients with fever ‘Fever (> 38*C*°)’ fail to achieve any statistically significant correlation with daily positive COVID cases as shown in table 3. The ‘Total Cough’ count, on the other hand, achieves a higher statistically significant correlation with postive COVID count (*ρ* = 0.40*, p* < 0.001). To compare the effect of these two measurements as feature in our COVID models, we used two different feature sets for training random forest-based model since it performed better than most of the other models we tried. Both of these sets contain the features ‘Total Speech’, ‘std energy’. The main difference is in the third feature which is ‘Fever(*>* 38C)’ and ‘Total cough’ respectively for the two sets. We can see from Table 7 that models with the ‘Total cough’ feature perform notably better than models with ‘Fever(> 38C)’ for the three target variables across all the evaluation metrics.

### Feature Importance Analysis

**Fig. 4:**
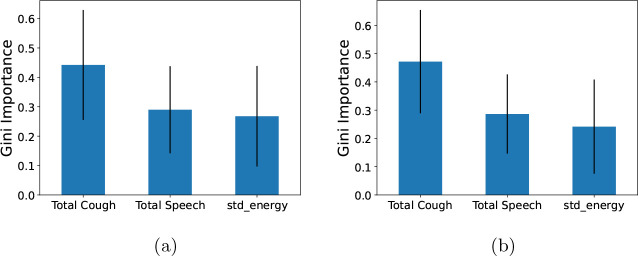
Gini feature importance for COVID burden estimation models: (a) modeling total Positive COVID cases and (b) modeling COVID-positive cases as fraction of the number of people

We analyze the importance of the features in our best performing models to get a clear idea of which features play the most important role in the model prediction. Figure 4 shows Gini feature importance in our models for predicting COVID related statistics. We can observe that ‘Total Cough’ has the highest importance for predicting both positive COVID count and positive COVID count as fraction of total number people visiting the hospital. This shows the prominence of total cough as feature in the models.

**Fig. 5:**
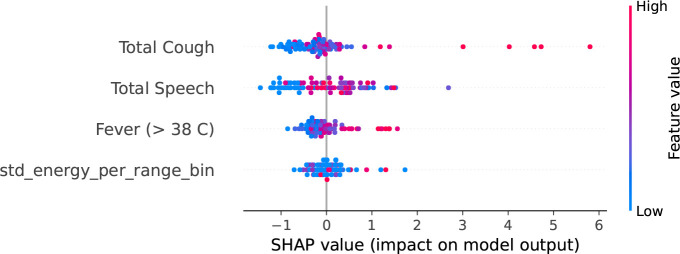
SHAP summary plot showing impact of feature values on model output

**Fig. 6:**
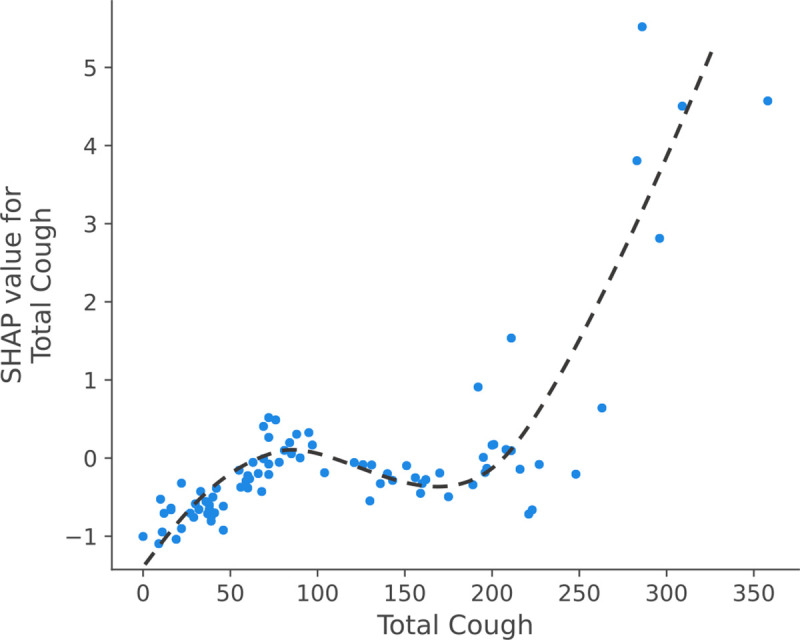
SHAP value for Total Cough feature

To compare with body temperature-based feature, we trained a Random Forest model using four specific features: ‘Total Cough’, ‘Total Speech’, ‘std energy’, and ‘Fever (> 38°C)’, to predict total COVID cases. To demonstrate the impact of features on model output, we utilized SHapley Additive exPlanations (SHAP) with TreeExplainer [13]. SHAP analysis helps determine how individual feature values impact the output of a model. As depicted in Figure 5, higher values of total cough correspond to increased model prediction output. We observe similar pattern for the ‘Fever (> 38°C)’ feature, however it contributes to a lesser extent compared to ‘Total Cough’. From Figure 6, we can observe that high values for the cough count correspond to increased SHAP values, indicating their importance in predicting positive COVID cases.

## Discussion

In our study, we identified crowd-level cough count as a robust indicator of the overall burden of COVID-19 cases. Our analysis revealed a significant and positive correlation between aggregated cough count and the total number of COVID-19 cases. Cough count exhibited a stronger correlation compared to other indicators, such as the count of individuals presenting with fever. We also observed a strong correlation between the total number of individuals visiting the ED waiting room and the count of positive COVID cases. We demonstrated how cough count, along with other signals from our *Syndromic Logger* platform, could be useful for modeling the daily COVID case counts by developing regression models. Notably, the inclusion of cough count as a feature yielded the highest model performance in terms of multiple evaluation metrics. Feature importance analysis of our models highlighted cough count as the most influential predictor, based on Gini importance and SHAP value metrics.

Our results contribute to the growing body of evidence on how syndromic surveillance can improve our resilience to emerging public health crises, such as the COVID-19 pandemic and potential future outbreaks of similar infectious diseases. Specifically, our study provides compelling evidence that monitoring aggregated cough count serves as a reliable syndromic signal, which has the potential to complement existing population-level surveillance methods. While body temperature screening has been widely adopted in hospitals and public spaces as a preventive measure against the spread of COVID-19 and similar diseases, our analysis reveals a stronger correlation between COVID burden and aggregated cough count compared to the count of individuals with fever. This underscores the significance of monitoring such signals, which can be accomplished in a contactless and unobtrusive manner using our *Syndromic Logger* platform. By highlighting the association between aggregated cough count and COVID-19 burden, our finding emphasizes the importance of incorporating this signal into public health surveillance strategies which is consistent with previous findings such as [3].

The monitoring of cough count holds significant relevance as a syndromic signal, and several key advantages make it highly advantageous to track. The measurement of cough count can be done with very high accuracy using machine learning-based models, which we discuss in detail in the methods section. With this capability, monitoring cough count provides a more objective signal compared to other signals such as trends in search engines or social media websites. Unlike the aforementioned signals, monitoring of cough offers several distinct advantages. It can be achieved without requiring active human participation or active effort. Our proposed approach enables real-time insights without any reporting delays. Additionally, we prioritize the security of the captured data and the preservation of privacy by collecting only aggregated information. One limitation of our approach relates to the cost associated with the initial deployment of the sensor platforms and the occasional maintenance these may require. However, this is not a major constraint since we primarily utilize low-cost sensors, and similar costs are typically associated with other syndromic surveillance approaches. Of note, the sensor platform was deployed in a hospital emergency room for the scope of this study. Consequently, our analysis does not encompass data from other non-hospital locations since we collected ground truth information from the hospital’s electronic health records (EHR). This study design allowed us to leverage reliable and precise data sources, which contributed to ensuring the accuracy of our findings.

Limitations may include the acoustic environment of this specific hospital setting; this specific patient populations which may differ from populations elsewhere; and the predominant virus during the study timeframe (delta; https://covariants.org/per-country?region=United+States) may have a differing syndromic profile from other SARS-CoV-2 variants [4] [15].

Based on our findings, we propose the integration of a sensor-based, contactless, and unobtrusive platform as a valuable augmentation to existing approaches, such as body temperature-based screening, in diverse public environments. Our results demonstrate that sensor-based platforms have the ability to capture clinically relevant syndromic signals, specifically the aggregated cough count. This capability allows us to overcome the limitations of existing methods which include reliance on manual human logging and reporting, as well as associated reporting delays. By embracing the adoption of sensor-based platforms, we can enhance the effectiveness and efficiency of public health surveillance, providing a superior solution for detecting and monitoring public health situations to overcome potential challenges in the future.

## Figures and Tables

**Fig. 3:**
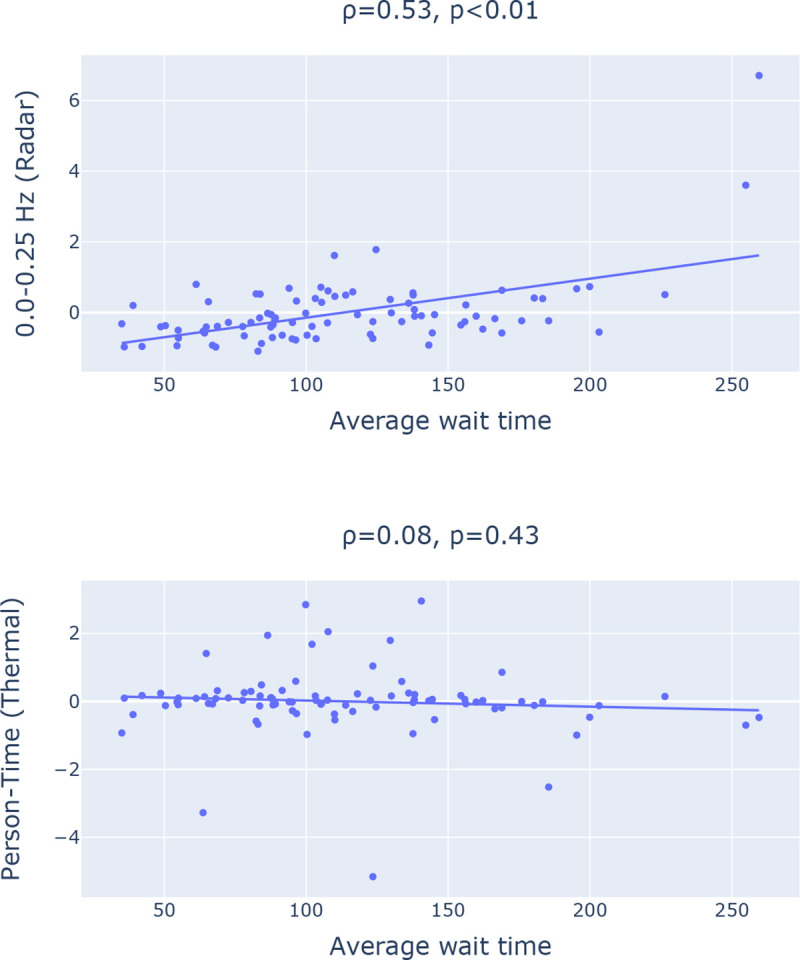
Scatter plot highlighting Pearson Correlation between Average wait time (from EHR) and two features extracted from respectively radar and thermal camera.

**Table 1:** Performance of our cough model in different settings

Testing sound type	VGGish model			FluSense model [3]		
	*R (%)*	*P (%)*	*F1 (%)*	*R (%)*	*P (%)*	*F1 (%)*
No Background Noise	91.5	91.5	91.5	90.2	90.2	90.2
With Speech	87	85.5	86	82.4	82.3	82.4
With Hospital Noise	87	88	86	84.5	85.4	84.4
With All Augmentations	89.5	90.5	89.5	87	87.3	86.9
From FluSense [3]	93.1	93.2	93	89	87	88

**Table 2:** Pearson Correlation between different syndromic features and Positive SARS-CoV-2, Influenza and RSV counts; p-value in parenthesis.

Modality	Features	Positive COVID	Positive Flu	Positive RSV

Audio	Total Cough	**0.40(<0.001)**	−0.11(0.30)	**0.27(0.01)**
	Total Speech	0.20(0.06)	0.03(0.80)	0.00(1.00)

Thermal	Person-Time	−0.06(0.56)	0.00(0.98)	−0.07(0.53)

	num_peaks	−0.20(0.06)	0.25(0.02)	0.05(0.63)
	*std_energy*	0.18(0.10)	0.23(0.03)	0.19(0.07)
Radar	tot_energy	0.15(0.15)	−0.23(0.03)	0.17(0.12)
	0.0–0.25 Hz	0.13(0.23)	−0.16(0.14)	0.13(0.21)
	0.25–0.50 Hz	0.13(0.22)	−0.23(0.03)	0.15(0.15)
	2.50–2.75 Hz	0.15(0.16)	−0.23(0.03)	0.17(0.13)

**Table 3:** Pearson correlation coefficient between different infections and waiting room crowd-related variables extracted from the hospital EHR systems. The p-value is reported within the parenthesis.

	# of People	Fever(> 38 C)	Positive COVID	Positive Flu

# of People	-			
Fever(> 38 C)	**0.45(<0.001)**	-		
Positive COVID	**0.28(0.01)**	0.22 (0.04)	-	
Positive Flu	0.04(0.72)	0.15 (0.16)	−0.02(0.86)	-
Positive RSV	0.07(0.51)	0.07(0.51)	**0.38(<0.001)**	−0.17(0.12)

**Table 4:** Multivariable negative binomial model quantifying relationship between SARS-CoV-2 positive tests, and cough-based syndromic signals, Massachusetts USA, 2021–22. (Note: IRR = incidence rate ratio (exponentiated coefficients).

Exploratory factor	IRR	95% CI	p-value
Total Cough per loge(0.25–0.50 Hz) signal	1.13	1.06 to 1.20	<0.001
**Day of Week**			
Sunday	(ref)	-	-
Monday	1.73	1.04 to 2.88	0.036
Tuesday	1.45	0.85 to 2.49	0.174
Wednesday	1.51	0.89 to 2.57	0.130
Thursday	1.43	0.84 to 2.44	0.188
Friday	1.33	0.78 to 2.29	0.296
Saturday	1.46	0.87 to 2.47	0.155

**Table 5:** List of features in baseline and sensor-based feature sets

Feature Set	List of Features

B1	Day of Week
B2	Day of Week, Positive COVID (previous 1 day)
B3	Day of Week, Positive COVID (previous 2 days)
B4	Day of Week, Positive COVID (previous 3 days)
S1	Day of Week, 0.0–0.25 Hz, Total Cough
S2	Total Cough, Total Speech, std_energy

**Table 6:** Leave one day out cross-validation results for different models with baseline and sensor-based features for three target variables: total COVID-positive cases, COVID cases as a percentage of hospital visits, and number of tests done

Model	Feature Set	Total COVID		COVID / People ratio (%)		COVID / Test ratio (%)	
		MAE	Pearson corr	MAE	Pearson corr	MAE	Pearson corr
Random Forest	B1	2.1	−0.24	0.95	−0.32	4.63	−0.25
	B2	2.08	0.25	0.89	0.25	4.85	−0.02
	B3	2.2	0.19	0.96	0.17	5.09	−0.1
	B4	2.14	0.13	0.92	0.16	4.55	0.02
	S1	1.8	0.41	0.76	0.47	4.30	0.26
	S2	1.78	0.43	0.75	0.49	3.65	0.38
Linear Regression	S2	1.91	0.29	0.82	0.34	3.99	0.24
Poisson Regression	S2	1.89	0.29	0.8	0.34	3.99	0.22
Grad-Boost	S2	1.77	0.45	0.75	0.53	3.66	0.43
SVR	S2	1.66	0.42	0.76	0.37	3.86	0.36

**Table 7:** Leave one day out cross-validation results with Random Forest model with two different feature sets; fs1 indicates {‘Fever(> 38C)’, ‘Total Speech’, ‘std_energy’} and fs2 indicates {‘Total cough’, ‘Total Speech’, ‘std_energy’}

Target variable	MAE		RMSE		Pearson corr	
	fs1	fs2	fs1	fs2	fs1	fs2
Positive COVID (#)	2.26	1.78	3.44	2.53	0.03	0.43
COVID/People ratio(%)	0.96	0.75	1.47	1.05	0.05	0.49
COVID/Test ratio(%)	4.5	3.65	6.13	5.2	0.15	0.38

## Data Availability

De-identified data will be made freely available, to qualified academic investigators for non-commercial research as required by the National Science Foundation and National Institutes of Health (NIH) Grants Policy on Sharing of Unique Research Resources and as permitted by the UMass IRB. Investigators must submit a formal request for data to the Principal Investigator (trahman@ucsd.edu) who will grant permission to release the data as long as it meets the following requirements: (1) institution-specific permission to use the data for research, (2) guarantee that data will be used for research purposes only, and (3) completion of a standard data use agreement.

## References

[1] 2022. FluSense-data. https://github.com/Forsad/FluSense-data

[2] Ageron Francois-Xavier, Hugli Olivier, Dami Fabrice, Caillet-Bois David, Pittet Valerie, Eckert Philippe, Beysard Nicolas, and Carron Pierre-Nicolas. 2022. Lessons from COVID-19 syndromic surveillance through emergency department activity: a prospective time series study from western Switzerland. BMJ open 12, 5 (2022), e054504. 10.1136/bmjopen-2021-054504 PMC9082728 35523491

[3] Al Hossain Forsad, Lover Andrew A., Corey George A., Reich Nicholas G., and Rahman Tauhidur. 2020. FluSense: A Contactless Syndromic Surveillance Platform for Influenza-Like Illness in Hospital Waiting Areas. Proc. ACM Interact. Mob. Wearable Ubiquitous Technol. 4, 1, Article 1 (March 2020), 28 pages. 10.1145/3381014 PMC9286491 35846237

[4] Flury Baharak Babouee, Güsewell Sabine, Egger Thomas, Leal Onicio, Brucher Angela, Lemmenmeier Eva, Kleeb Dorette Meier, Möller J Carsten, Rieder Philip, Rütti Markus, 2022. Risk and symptoms of COVID-19 in health professionals according to baseline immune status and booster vaccination during the Delta and Omicron waves in Switzerland—A multicentre cohort study. PLoS medicine 19, 11 (2022), e1004125. 36342956 10.1371/journal.pmed.1004125 PMC9678290

[5] Gemmeke Jort F., Ellis Daniel P. W., Freedman Dylan, Jansen Aren, Lawrence Wade, Moore R. Channing, Plakal Manoj, and Ritter Marvin. 2017. Audio Set: An ontology and human-labeled dataset for audio events. In Proc. IEEE ICASSP 2017. New Orleans, LA.

[6] Güemes Amparo, Ray Soumyajit, Aboumerhi Khaled, Desjardins Michael R, Kvit Anton, Corrigan Anne E, Fries Brendan, Shields Timothy, Stevens Robert D, Curriero Frank C, 2021. A syndromic surveillance tool to detect anomalous clusters of COVID-19 symptoms in the United States. Scientific reports 11, 1 (2021), 4660. 33633250 10.1038/s41598-021-84145-5 PMC7907397

[7] Harris Paul A, Taylor Robert, Thielke Robert, Payne Jonathon, Gonzalez Nathaniel, and Conde Jose G. 2009. Research electronic data capture (REDCap)—a metadata-driven methodology and workflow process for providing translational research informatics support. Journal of biomedical informatics 42, 2 (2009), 377–381. 18929686 10.1016/j.jbi.2008.08.010 PMC2700030

[8] Hershey Shawn, Chaudhuri Sourish, Ellis Daniel P. W., Gemmeke Jort F., Jansen Aren, Moore Channing, Plakal Manoj, Platt Devin, Saurous Rif A., Seybold Bryan, Slaney Malcolm, Weiss Ron, and Wilson Kevin. 2017. CNN Architectures for Large-Scale Audio Classification. In International Conference on Acoustics, Speech and Signal Processing (ICASSP). https://arxiv.org/abs/1609.09430

[9] Hughes Helen E, Hughes Thomas C, Morbey Roger, Challen Kirsty, Oliver Isabel, Smith Gillian E, and Elliot Alex J. 2020. Emergency department use during COVID-19 as described by syndromic surveillance. Emergency Medicine Journal 37, 10 (2020), 600–604. 32948621 10.1136/emermed-2020-209980 PMC7503196

[10] Ijaz Aneeqa, Nabeel Muhammad, Masood Usama, Mahmood Tahir, Hashmi Mydah Sajid, Posokhova Iryna, Rizwan Ali, and Imran Ali. 2022. Towards using cough for respiratory disease diagnosis by leveraging Artificial Intelligence: A survey. Informatics in Medicine Unlocked 29 (2022), 100832. 10.1016/j.imu.2021.100832

[11] Lapointe-Shaw Lauren, Rader Benjamin, Astley Christina M, Hawkins Jared B, Bhatia Deepit, Schatten William J, Lee Todd C, Liu Jessica J, Ivers Noah M, Stall Nathan M, 2020. Web and phone-based COVID-19 syndromic surveillance in Canada: A cross-sectional study. PLoS One 15, 10 (2020), e0239886. 33006990 10.1371/journal.pone.0239886 PMC7531838

[12] Lauer Stephen A, Grantz Kyra H, Bi Qifang, Jones Forrest K, Zheng Qulu, Meredith Hannah R, Azman Andrew S, Reich Nicholas G, and Lessler Justin. 2020. The incubation period of coronavirus disease 2019 (COVID-19) from publicly reported confirmed cases: estimation and application. Annals of internal medicine 172, 9 (2020), 577–582. 32150748 10.7326/M20-0504 PMC7081172

[13] Lundberg Scott M., Erion Gabriel, Chen Hugh, DeGrave Alex, Prutkin Jordan M., Nair Bala, Katz Ronit, Himmelfarb Jonathan, Bansal Nisha, and Lee Su-In. 2020. From local explanations to global understanding with explainable AI for trees. Nature Machine Intelligence 2, 1 (2020), 2522–5839. 10.1038/s42256-019-0138-9 PMC7326367 32607472

[14] Maltezou Helena C, Papanikolopoulou Amalia, Vassiliu Sofia, Theodoridou Kalliopi, Nikolopoulou Georgia, and Sipsas Nikolaos V. 2023. COVID-19 and Respiratory Virus Co-Infections: A Systematic Review of the Literature. Viruses 15, 4 (2023), 865. 37112844 10.3390/v15040865 PMC10142898

[15] Marquez Carina, Andrew D Kerkhoff John Schrom, Rojas Susana, Black Douglas, Mitchell Anthea, Wang Chung-Yu, Pilarowski Genay, Ribeiro Salustiano, Jones Diane, 2022. COVID-19 Symptoms and Duration of Rapid Antigen Test Positivity at a Community Testing and Surveillance Site During Pre-Delta, Delta, and Omicron BA. 1 Periods. JAMA Network Open 5, 10 (2022), e2235844–e2235844. 36215069 10.1001/jamanetworkopen.2022.35844 PMC9552893

[16] Nomura Shuhei, Yoneoka Daisuke, Shi Shoi, Tanoue Yuta, Kawashima Takayuki, Eguchi Akifumi, Matsuura Kentaro, Makiyama Koji, Ejima Keisuke, Taniguchi Toshibumi, 2020. An assessment of self-reported COVID-19 related symptoms of 227,898 users of a social networking service in Japan: Has the regional risk changed after the declaration of the state of emergency? The Lancet Regional Health-Western Pacific 1 (2020), 100011. 34173594 10.1016/j.lanwpc.2020.100011 PMC7453215

[17] Osthus Dave, Daughton Ashlynn R., and Priedhorsky Reid. 2019. Even a good influenza forecasting model can benefit from internet-based now-casts, but those benefits are limited. PLOS Computational Biology 15, 2 (02 2019), 1–19. 10.1371/journal.pcbi.1006599 PMC6373968 30707689

[18] Porter Paul, Abeyratne Udantha, Swarnkar Vinayak, Tan Jamie, Ng Ti-wan, Brisbane Joanna M, Speldewinde Deirdre, Choveaux Jennifer, Sharan Roneel, Kosasih Keegan, 2019. A prospective multicentre study testing the diagnostic accuracy of an automated cough sound centred analytic system for the identification of common respiratory disorders in children. Respiratory research 20, 1 (2019), 1–10. 31167662 10.1186/s12931-019-1046-6 PMC6551890

[19] Ren Shaoqing, He Kaiming, Girshick Ross, and Sun Jian. 2015. Faster R-CNN: Towards Real-Time Object Detection with Region Proposal Networks. In Advances in Neural Information Processing Systems, Cortes C., Lawrence N., Lee D., Sugiyama M., and Garnett R. (Eds.), Vol. 28. Curran Associates, Inc. https://proceedings.neurips.cc/paper_files/paper/2015/file/14bfa6bb14875e45bba028a21ed38046-Paper.pdf

[20] Ribeiro Miguel, Teixeira Diogo, Barbosa Pedro, and Nunes Nuno Jardim. 2023. Using passive Wi-Fi for community crowd sensing during the COVID-19 pandemic. Journal of Big Data 10, 1 (2023), 1–22. 36686620 10.1186/s40537-022-00675-3 PMC9844951

[21] Simonyan Karen and Zisserman Andrew. 2014. Very Deep Convolutional Networks for Large-Scale Image Recognition. 10.48550/ARXIV.1409.1556

[22] Swets Maaike C, Russell Clark D, Harrison Ewen M, Docherty Annemarie B, Lone Nazir, Girvan Michelle, Hardwick Hayley E, Visser Leonardus G, Openshaw Peter JM, Groeneveld Geert H, 2022. SARS-CoV-2 co-infection with influenza viruses, respiratory syncytial virus, or adenoviruses. The Lancet 399, 10334 (2022), 1463–1464. 10.1016/S0140-6736(22)00383-X PMC8956294 35344735

[23] Ting Daniel Shu Wei, Carin Lawrence, Dzau Victor, and Wong Tien Y. 2020. Digital technology and COVID-19. Nature medicine 26, 4 (2020), 459–461. 10.1038/s41591-020-0824-5 PMC7100489 32284618

[24] Yom-Tov Elad. 2021. Active syndromic surveillance of COVID-19 in Israel. Scientific Reports 11, 1 (2021), 24449. 34961786 10.1038/s41598-021-03977-3 PMC8712517

